# Global Proteome of *LonP1*^+/−^ Mouse Embryonal Fibroblasts Reveals Impact on Respiratory Chain, but No Interdependence between Eral1 and Mitoribosomes

**DOI:** 10.3390/ijms20184523

**Published:** 2019-09-12

**Authors:** Jana Key, Aneesha Kohli, Clea Bárcena, Carlos López-Otín, Juliana Heidler, Ilka Wittig, Georg Auburger

**Affiliations:** 1 Experimental Neurology, Goethe University Medical School, 60590 Frankfurt am Main, Germany; key@stud.uni-frankfurt.de (J.K.); aneeshak19@gmail.com (A.K.); 2 Departamento de Bioquimica y Biologia Molecular, Facultad de Medicina, Instituto Universitario de Oncologia (IUOPA), Universidad de Oviedo, 33006 Oviedo, Spain; cleabarcena@gmail.com (C.B.); clo@uniovi.es (C.L.-O.); 3 Functional Proteomics Group, Goethe-University Hospital, 60590 Frankfurt am Main, Germany; jheidler@em.uni-frankfurt.de

**Keywords:** longevity, life expectancy, CODAS syndrome, Perrault syndrome, protease target substrates, respiratory complex assembly, oxidative stress, glutathione pathway, lysosomal degradation, fidelity protein synthesis

## Abstract

Research on healthy aging shows that lifespan reductions are often caused by mitochondrial dysfunction. Thus, it is very interesting that the deletion of mitochondrial matrix peptidase LonP1 was observed to abolish embryogenesis, while deletion of the mitochondrial matrix peptidase Caseinolytic Mitochondrial Matrix Peptidase Proteolytic Subunit (ClpP) prolonged survival. To unveil the targets of each enzyme, we documented the global proteome of *LonP1*^+/−^ mouse embryonal fibroblasts (MEF), for comparison with *ClpP*^−/−^ depletion. Proteomic profiles of *LonP1*^+/−^ MEF generated by label-free mass spectrometry were further processed with the STRING (Search tool for the retrieval of interacting genes) webserver Heidelberg for protein interactions. ClpP was previously reported to degrade Eral1 as a chaperone involved in mitoribosome assembly, so ClpP deficiency triggers the accumulation of mitoribosomal subunits and inefficient translation. *LonP1*^+/−^ MEF also showed Eral1 accumulation, but no systematic effect on mitoribosomal subunits. In contrast to *ClpP*^−/−^ profiles, several components of the respiratory complex-I membrane arm, of the glutathione pathway and of lysosomes were accumulated, whereas the upregulation of numerous innate immune defense components was similar. Overall, LonP1, as opposed to ClpP, appears to have no effect on translational machinery, instead it shows enhanced respiratory dysfunction; this agrees with reports on the human CODAS syndrome (syndrome with cerebral, ocular, dental, auricular, and skeletal anomalies) caused by LonP1 mutations.

## 1. Introduction

Mitochondria are double membraned subcellular organelles which are implicated in multiple cellular processes. In addition to their main role in the production of energy through being the organelle housing oxidative phosphorylation and the citric acid cycle, mitochondria exert many other functions. These range from cellular calcium homeostasis [1], regulation of reactive oxygen species (ROS), assembly of iron–sulfur-cluster and heme, to participation in innate immune responses of any cell type. Respecting their manifold roles mitochondria have been shown to play important roles in various diseases, such as neurodegenerative diseases [2], inflammatory damage [3,4], and cancer [5,6].

Within mammalian mitochondria, four energy (ATP)-dependent proteolytic systems exist, with Lonp1 (Lon Protease Homolog 1) in the matrix being the simplest example as a soluble homo-hexamer single-ring with each subunit containing an AAA+ (ATPases Associated with diverse cellular Activities) ATPase plus a serine peptidase domain. In contrast, ClpXP protease in the matrix is also soluble but consists of two stacked heptameric ClpP (Caseinolytic Mitochondrial Matrix Peptidase Proteolytic Subunit) serine peptidase rings attached to caps of hexameric ClpX (Caseinolytic Mitochondrial Matrix Peptidase Chaperone Subunit) AAA+ ATPase rings at either end. In the mitochondrial inner membrane, there are two proteolytic machines with AAA+ ATPase domains and zinc metalloproteinase catalytic complexes plus transmembrane domains in opposite orientation: With orientation towards the matrix, the m-AAA protease consists of an Afg3l2 homo-hexameric ring or an Afg3l2/Sph7 hetero-hexameric ring. With orientation towards the intermembrane space, the i-AAA protease consists of a Ymel1 hexameric ring [2,7,8]. Mutations in each of these four ATP-dependent protease complexes invariably lead to nervous system atrophy, which may manifest as psychomotor delay, hypomyelination, ataxia, spasticity, or neuropathy [9,10,11,12].

Signals that reflect any deterioration of mitochondrial health arise through intramembrane proteolysis by Parl, the rhomboid-like serine protease associated with cleavage of the apoptosis factor Pgam5 [13,14] and the Parkinson’s disease protein Pink1. Its mutation affects mitochondrial turnover via Parkin-dependent mitophagy, where variants of hereditary Parkinsonism named PARK6 and PARK2 are triggered [1,3,15,16,17,18,19,20,21,22]. The Parkinsonism variant PARK7 is caused by mutations in DJ-1, a cysteine pseudoprotease that lacks key residues for catalysis but may regulate the activity of homologous proteases [8,23]. It was shown to relocalize to mitochondria upon oxidative stress and protects cells from the formation of α-synuclein aggregates as the principal cause of Parkinson’s disease [24,25].

Mitochondrial health has to be maintained by various quality control pathways. Among them, proteostasis is crucial, with close monitoring of precursor import, folding, complex assembly, unfolded protein responses and proteolytic degradation. When the amount of damage cannot be repaired via substitution of individual factors, mitochondrial fragments can be eliminated by fission and mitochondrial autophagy, or the whole cell can undergo apoptosis. Postmitotic neurons are particularly dependent on mitochondrial health and their loss cannot be substituted, so many mitochondrial dysfunctions result in neurological diseases [26].

Mitochondrial dysfunction often triggers a change of lifespan [27,28]. Using the survival of cells and organisms as criteria, it seems clear that LonP1 is the main mitochondrial matrix protease given that its deficiency results in embryonic lethality [6]. In contrast, ClpP seems to play an accessory role in stress response, with its deficiency even extending lifespan in some fungus named *Podospora anserina* [29].

LonP1 is an AAA+ domain containing, highly conserved serine peptidase, functioning as protease, chaperone and interactor of single-stranded mitochondrial DNA [30,31,32,33]. It has been shown to be responsible for the degradation of misfolded or damaged proteins and for the assembly of respiratory chain complexes [34]. Point mutations of LonP1 in humans lead to the development of a rare disease named CODAS syndrome (cerebral, ocular, dental, auricular, and skeletal syndrome) with failure of oxidative phosphorylation [12,35,36].

LonP1 has been described to degrade several proteins within the mitochondrial matrix, such as mitochondrial Aconitase (Aco2) [31], a Cytochrome *c* oxidase subunit (Cox4-1) [37], Mitochondrial Steroidogenic Acute Regulatory Protein (StAR) [38], the rate-limiting enzyme in heme biosynthesis (Alas1) [39], and the phosphorylated form of mitochondrial transcription factor A (Tfam) [40]. A systematic global survey of its degradation substrates, their half-life changes, and possible compensatory efforts has not been carried out.

ClpP in the mitochondrial matrix is also a highly conserved serine peptidase, which is assembled in a barrel-like structure together with ClpX, the latter one providing energy via its ATPase function and assuring substrate specificity [41]. ClpP has been shown to play a role in the unfolded protein response in mitochondria (UPRmt) in *C. elegans* and may play a role in the degradation of unfolded proteins in rodents as well as in humans [42,43,44]. Mutations in ClpP in humans lead to the development of Perrault syndrome 3 (PRLTS3), which is modeled by ClpP^−/−^ mice that mirror the infertility, sensorineural hearing loss, ataxia and growth retardation known from PRLTS3 patients [45,46]. In addition, mitochondrial DNA (mtDNA) content was shown to be present in excess in the absence of ClpP. It was recently shown that ClpP deficiency impairs the fidelity of mitochondrial translation and leads to an accumulation of mitoribosomes. This is thought to be caused by the pathological accumulation of the mitoribosomal assembly factor Eral1, which was claimed to be a direct proteolytic substrate of ClpP [47].

While the deficiency of LonP1 on the one hand and the subsequent accumulation of misfolded proteins in the mitochondria trigger an increase of Pink1 and the engulfment/elimination of mitochondria via the autophago–lysosomal pathway [48], the deficiency of ClpP, on the other hand, triggers selective changes in the cytosolic proteasome [46]. In both cases, the mitochondrial dysfunction caused by either LonP1 or ClpP depletion activates the Rig-I-like receptor pathway of the innate immune system, perhaps simply by altered binding of lactate and hexokinase‑2 to the mitochondrial antiviral signaling protein Mavs [46,49,50,51]. In order to elucidate the pathology of mitochondrial proteostasis and the compensatory efforts of the surrounding eukaryotic cell in further detail, we used *LonP1*^+/−^ mouse embryonic fibroblasts (MEFs) to document their global proteome profile. The comparison with previously reported *ClpP*^−/−^ expression profiles may help to identify substrates that are selectively degraded by each of these proteases.

## 2. Results

The analysis of previously generated [6] *LonP1*^+/−^ MEFs versus wildtype littermate MEFs regarding their global proteome profile by quantitative label-free mass spectrometry detected a total of 5929 proteins. A high number exhibited significant changes in abundance (Supplementary Table S1). Among them, 463 proteins were upregulated and 328 downregulated with nominal significance. Thus, despite the deficiency of the main mitochondrial protease in these cells, there was no strong bias towards protein accumulations in the profile. However, the effects were clearly non-random, with highly significant enrichments of specific pathways and an overall protein–protein-interaction enrichment *p*-value of <1.0 × 10^−16^ upon bioinformatics analysis at the STRING (search tool for the retrieval of interacting genes) webpage in Heidelberg. Interestingly, known LonP1 degradation targets such as Tfam and Aco2 [6,40,52] did not show significantly elevated abundance in this heterozygous LonP1 depletion state. It is also noteworthy that indicators of mitochondrial biogenesis such as Ppargc1a, Nrf1, Nrf2, Tfam [53], or components of the selective autophagy for mitochondria such as Pink1, Park2, Bnip3, Bnip3l, Fundc1, Ambra1, Mul1, Arih1, March5, gp78/Amfr, Mgrn1, and Huwe1 [54,55] did not show dysregulated abundance. With regard to the selectivity of proteolytic degradation by Lonp1 versus ClpXP in the mitochondrial matrix, it is important to note that mitoribosomal subunits were not accumulated in *LonP1*^+/−^ MEFs, in contrast to *ClpP*^−/−^ proteome profiles.

Among the upregulated factors, enrichment analyses with STRING highlighted the KEGG pathway of “lysosome” (false discovery rate FDR = 8.75 × 10^−12^) and the REACTOME pathways “neutrophil degranulation” (FDR = 1.08 × 10^−16^) and “innate immune system” (FDR = 1.94 × 10^−12^).

Among the downregulated factors, STRING enrichment analyses highlighted the KEGG pathway “focal adhesion” (FDR = 2.82 × 10^−12^), the REACTOME pathway “cell junction organization” (FDR = 4.21 × 10^−6^) and the INTERPRO domain features “zinc finger, LIM type” (FDR = 3.08 × 10^−6^).

**Figure 1:**
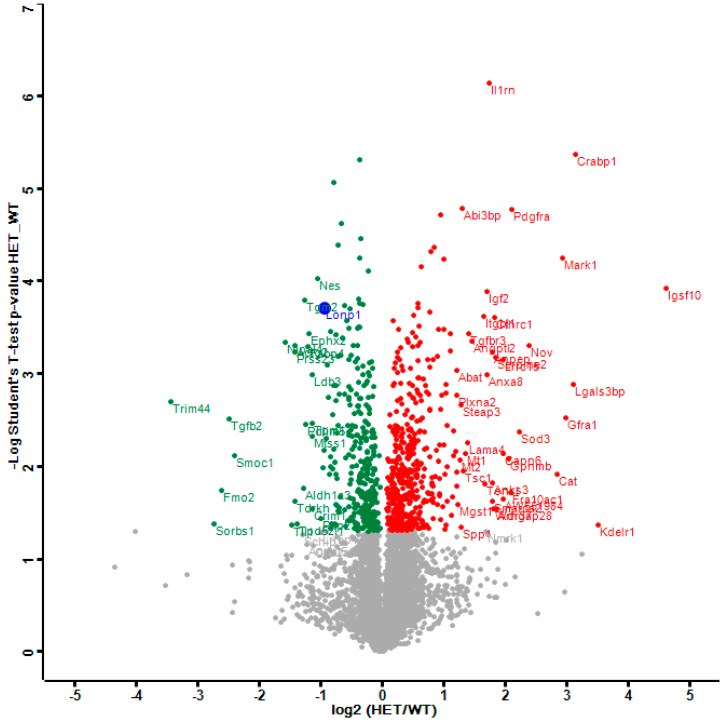
This Volcano plot represents all significant dysregulations in the global proteome of *LonP1*^+/−^ mouse embryonal fibroblasts (MEFs) (heterozygous mutant (HET)) in green color (downregulated), red color (upregulated) or blue color (genetically deleted). The two-fold deficiency of LonP1 is represented by log2 ratio as –1 on the X-axis. Significance values are shown on the Y-axis. Peptides with non-significant changes are shown as grey dots. Factors of high significance despite moderate fold-change such as LonP1 can be easily distinguished in this diagram from other factors of massive fold-changes with higher variability such as the longevity and health span factor Fmo2 [59].

The distribution of dysregulations and the most important factors are illustrated in a Volcano plot (Figure 1), where the genetically reduced abundance of LonP1 is illustrated with a blue dot, other significant downregulations are shown in green, and the relevant upregulations in red color. The strongest significant downregulation concerned Trim44, as a modulator of glycolysis together with lactate production, and as a modulator of inflammation via interaction with Mavs [50,56,57]. The strongest significant upregulations concerned several components of immune pathways such as Igsf1, Kdelr1, and Lgals3bp, but importantly, also a crucial defense factor against oxidative stress, namely Cat (catalase). Its induction probably has compensatory nature, given that it is known to protect mitochondrial energetics in the face of LonP1 deficiency [58].

Given that our further analyses focus on pathways with strong enrichment at the FDR significance level, we used less stringency for their individual components, referring to their nominal *p*-values.

### 2.1. Dysregulated Mitochondrial Factors in LonP1^+/−^ MEFs are Enriched for Oxidation Processes

To understand how LonP1 deficiency triggers these cellular responses, we selected among all significant dysregulations only those factors that are enriched in mitochondria, based on their classification in the STRING database and the localization predictions in the GeneCards database (see Table 1).

**Figure 2:**
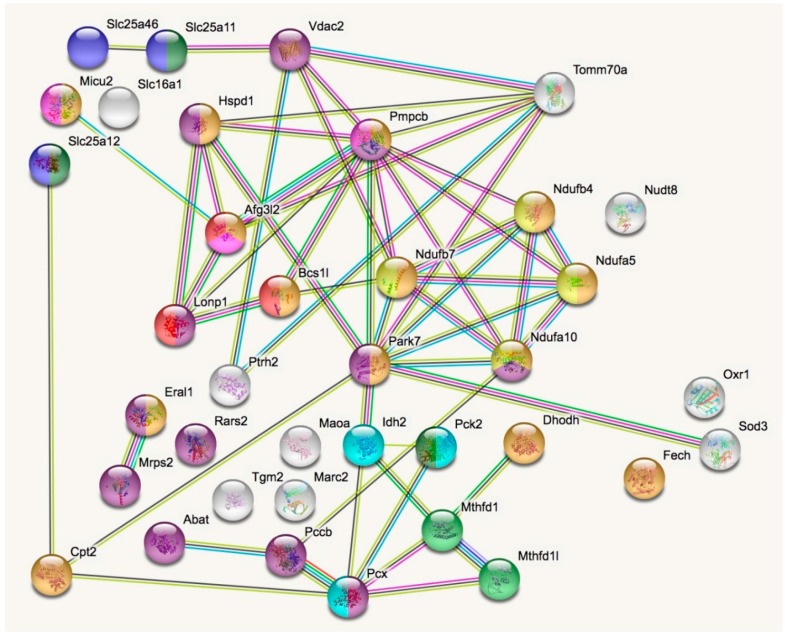
The STRING (Search tool for the retrieval of interacting genes) diagram of protein–protein interactions shows all significant dysregulations in mitochondria. In colors, it highlights mitochondrial matrix factors (violet), inner membrane proteins (orange), TCA cycle (Tricarboxylic acid cycle, Citric acid cycle, Krebs cycle) (light blue), gluconeogenesis (dark green), complex-I biogenesis (yellow), processing of Smdt1 (pink), formate-tetrahydrofolate ligase (light green), mitochondrial carrier domain superfamily (dark blue) and AAA+ ATPases (red). The membrane carriers and pore factors were manually placed in the left upper corner and upper margin, while the respiratory complex components were positioned in the right upper corner. LonP1 and its interaction with other disaggregases, proteases, and chaperones were located in the middle from left to center. The Eral1 mitoribosome chaperone and other translation components are shown towards the lower left corner, the breakdown of fatty acids/amino acids/one-carbon flux towards the lower center, and oxidative stress response in the lower right corner.

In a STRING analysis to identify regulated pathways within mitochondria (enrichment *p*-value <1.0 × 10^−16^), the changes concerned inner membrane factors (FDR = 3.81 × 10^−15^) even more strongly than the mitochondrial matrix factors (FDR = 3.37 × 10^−12^), in particular the Biological Process GO term “oxidation-reduction” (FDR = 5.33 × 10^−10^). Among the KEGG and REACTOME pathways, the “Complex-I biogenesis” (FDR = 5.14 × 10^−5^) and the “Processing of SMDT1” (FDR = 5.52 × 10^−5^) as inner membrane processes were affected more significantly than the matrix processes “citrate cycle” (FDR = 4.30 × 10^−4^) and gluconeogenesis (FDR = 2.50 × 10^−4^). Also in the matrix, the one-carbon metabolism was affected with “formate-tetrahydrofolate ligase” being prominent among PFAM protein domains (FDR = 9.60 × 10^−4^), while membrane processes again dominated with “mitochondrial carrier protein superfamily” (FDR = 2.0 × 10^−4^) among INTERPRO protein domains, and the dysregulation of several AAA+ disaggregases appeared as “ATPases associated with a variety of cellular activities” (FDR =2.09 × 10^−2^) among SMART protein domains, in response to deficiency of the AAA+ domain containing LonP1. Figure 2 illustrates these factors with various colors for each pathway, and with connecting lines that represent different evidence for interactions among them. Importantly, respiratory complex I subunits in the ND5, ND4 and ND2 module of the membrane arm accumulate, in contrast to a subunit from the Q module in the hydrophilic peripheral arm in the mitochondrial matrix. Overall, complex-I assembly stoichiometry appears to be more strongly affected by LonP1 deficiency than the previously reported LonP1-dependent degradation substrates such as Tfam or Aco2.

#### 2.1.1. Mitochondrial Downregulations in LonP1^+/−^ MEFs

In parallel with the deficiency of LonP1, strongly significant reductions appeared (Table 1) for the chaperone Hspd1, which is responsible of folding mitochondrial proteins to their native state [61], and for the peptidase Pmpcb (beta-MPP), which catalyzes the cleavage of mitochondrial precursor proteins upon import, before they are folded [62,63]. In addition, a strongly significant reduction of the Tomm70a import receptor in the outer membrane translocase suggests that the recruitment and folding of precursor proteins decrease notably. Three components of the nucleobase biosynthetic process were also diminished with strong significance, namely Dhodh as the only mitochondrial factor involved in *de novo* pyrimidine synthesis, as well as Mthfd1l and Mthfd1 that represent mitochondrial factors involved in de novo purine synthesis. These data suggest that RNA and DNA processes in LonP1^+/−^ mitochondria become unbalanced.

#### 2.1.2. Mitochondrial Upregulations in LonP1^+/−^ MEFs

The most prominent increase (p = 9.25 × 10^−4^) among mitochondrial factors (Table 1) concerned the transaminase Abat, which cooperates with the succinate-CoA ligases Suclg1 and Sucla2 to convert dNDPs to dNTPs, while also affecting the glutamate–glutamine cycle and mediating the GABA-shunt as a complement to the citric acid cycle that is crucial for anoxia and acid tolerance [64,65,66]. The second most significant upregulation was noted for the mitoribosome assembly factor Eral1. This was a surprising finding, given that Eral1 was recently claimed to be a selective substrate of ClpXP-mediated degradation [47]. ClpP protein levels were unchanged in LonP1^+/−^ MEF (Supplementary Table S1), ruling out that this effect was still dependent on ClpP. In a subsequent paragraph, we performed meta-analyses of two published proteome profiles in comparison to this proteome profile to assess the questions, whether mitoribosome accumulation is a ClpXP-specific effect, whether Eral1 accumulation is a ClpXP-specific effect (or also depends on the presence of LonP1), and whether mitoribosome and Eral1 levels depend on each other.

Several other upregulated factors in *LonP1*^+/−^ MEFs reflect enhanced oxoacid metabolism in the mitochondrial matrix, namely Pcx, Pck2, Pccb, Idh2, Cpt2, Marc2, and Rars2. The increased levels of Fech and Oxr1 presumably imply efforts to compensate oxidative stress within mitochondria. Importantly, the deficiency of the AAA+ domain containing peptidase LonP1 triggered elevated levels for two factors with a role in protein processing: Firstly, Bcsl1 as an AAA+ domain-containing assembly factor of respiratory complex-III [67] showed significant accumulation. Secondly, an accumulation was found for the AAA+ domain containing peptidase Afg3l2 (mAAA-subunit-2), which modulates assembly of respiratory complex-IV and of the MCU complex [68,69]. Both are highlighted in yellow in Table 1. Overall, many upregulations refer to oxoacid metabolism, oxidative stress, and assembly of the oxidative phosphorylation/respiratory chain.

#### 2.1.3. Eral1 and Mitoribosomal Factors as Substrates of LonP1 Versus ClpXP—Two Meta-Analyses

In an effort to understand, to what degree the degradation of the RNA-chaperone Eral1 is due to LonP1 or ClpXP, and whether Eral1 accumulation is prominently responsible for the assembly of the 28S ribosomal subunit in mitochondria, we reassessed previously published global proteome data from the heart of *ClpP^−/−^* mice [47]. Highly significant peptide accumulations were observed not only for Eral1, but also for several other mitochondrial matrix chaperones. In particular, a massive increase was observed for ClpX (91.8-fold, p = 3.8 × 10^−5^). Substantially elevated levels existed also for Trap1 (4.8-fold, p = 7.11 × 10^−6^), Grpel1 (3.5-fold, p = 7.03 × 10^−6^), Hspa9 (2.5-fold, p = 1.4 × 10^−5^), and Dnaja3 (2.1-fold, p = 3.6 × 10^−4^). It remained unclear to what degree excessive transcriptional induction versus deficient proteolytic turnover is responsible for these chaperone accumulations [47]. Thus, a host of chaperones show elevated levels in parallel to the mitoribosome accumulation.

In view of the massive ClpX accumulation above, it is also interesting to re-assess a recent study where ClpX had been overexpressed 1.8-fold in C2C12 mouse myoblast cells and the global proteome profile was documented [42]. While ClpX is elevated in both experiments, ClpP as an interactor protein showed a 1.4-fold co-accumulation upon ClpX overexpression, in contrast to the previous ClpP deletion study. Thus, a comparison of both profiles permits the identification of selective consequences of ClpP deficiency versus ClpP overactivity, separating them from indirect effects. We performed an analogous bioinformatics assessment of this published dataset, selecting the proteins with >1.5-fold accumulation and testing their pathway interaction profile in STRING enrichment statistics and diagrams. Indeed, the STRING diagram of upregulations upon ClpX overexpression (Supplementary Figure S1) detects similarly strong accumulations of mitoribosomal subunits as in *ClpP*^−/−^ tissues with their massive ClpX accumulation. Thus, strong mitoribosomal accumulation occurs in both cases together with elevated ClpX levels. In comparison, Eral1 levels showed only 1.1-fold upregulation in ClpX overexpression myoblasts. The most significant findings in ClpX overexpression myoblasts (see Supplementary Table S2) reflect mitochondrial and nucleolar anomalies, with altered ribosome biogenesis. Relevant similarities include the co-accumulation of Noa1 (1.7-fold), Hspa9 (1.5-fold), and Grpel2 (1.4-fold) with ClpX. The increased levels of many chaperones might simply be due to co-accumulation with misfolded mitochondrial ribosome complexes. Specifically, Eral1 may simply show retarded turnover upon the accumulation of its binding partners, mitoribosomes and RNAs. Overall, the available data suggest that the accumulation of mitoribosomes in *ClpP^−/−^* tissues occurs when ClpX levels are elevated and is independent from Eral1 accumulation. In contrast to mitoribosomes, Eral1 levels are not determined by ClpXP-mediated degradation alone, but elevated in ClpP deficiency, in ClpX overexpression, and in LonP1 deficiency.

### 2.2. Oxidative Stress and Glutathione Pathways in LonP1^+/−^ MEFs

Beyond the mainly mitochondrial factors, relevant enrichments among GO terms Biological processes were noted for “response to oxidative stress” (FDR = 0.00060) and “glutathione metabolic process” (FDR = 0.00055) in the LonP1^+/−^ MEF proteome profile, with an upregulation for each of these enzymes except the glutathione synthase Gss, the spermine synthase Sms and the hypoxia-inducible factor Egln1 (Table 2). These upregulations were strongest for Cat and Mgst1, which are present both in mitochondria and the cytosol and small for Gstm5 and Gstm2 whose mitochondrial presence is weak, according to localization predictions in the GeneCards database. It seems likely that these changes are secondary to the altered oxidative processes within mitochondria.

### 2.3. Activation of the Innate Immune System in LonP1^+/−^ MEFs

In the STRING analysis of all enrichments among REACTOME pathways, “neutrophil degranulation” (FDR = 3.16 × 10^−15^) and “innate immune system” (FDR = 4.26 × 10^−11^) were prominent. Among the individual dysregulations, the strongest effect was an ~11-fold downregulation of Trim44 (Table 3), which is responsible for stabilizing Mavs as the mitochondrial antiviral signaling factor [57]. Weaker downregulations concerned several Cluster of Differentiation (CD) antigens, namely CD44, CD97, CD109, and CD302. This is in good agreement with recent observations that dysfunctional mitochondria will release toxic dsRNA and mtDNA [70,71]. Indeed, within the innate immune system, there was an upregulation of cytosolic sensor pathway components, both for the detection of toxic RNA (namely Ifi35, Ifih1, Tspan6, Eif2ak2, and Trim25) and toxic DNA (Ifi204, Tmem173/STING, Mnda). Examining the subset of immunity-related dysregulations in a further STRING analysis, significant enrichments were detected for “response to cytokine” (FDR = 5.01 × 10^−7^), “defense response to virus” (FDR = 8.94 × 10^−7^), “Jak-Stat signaling pathway” (FDR = 0.00044), “NOD-like receptor signaling pathway” (FDR = 0.00044) and “RIG-I-like receptor signaling pathway” (FDR = 0.00044).

### 2.4. The Strongest Upregulations in LonP1^+/−^ MEFs can be Seen in the Lysosomal Pathway

In the STRING analysis of all enrichments, “lysosome” was prominent among KEGG pathways (FDR = 1.19 × 10^−18^), also among UNIPROT keywords (FDR = 1.18 × 10^−9^) and among GO terms Molecular Function (FDR = 5.27 × 10^−9^). Almost all dysregulations consisted of increased abundance of lysosomal factors, suggesting elevated degradation activity (Table 4). This overactivity seemed directed towards specific targets, given that the strongest upregulations concerned the lysosomal cathepsin Ctsc as an activator of many serine proteases in the immune system, and lysosomal cathepsin Ctsz also as a component of innate immune responses. Conversely, the strongest downregulation concerned the lysosomal cathepsin Ctsh, which is responsible for the overall degradation of proteins in lysosomes. While there was a strong increase for Tmem59, which directs endosomal LC3 labelling and lysosomal targeting, there was a reciprocal decrease for Sqstm1/p62, which directs protein aggregates to autophago-lysosomal degradation. It is also noteworthy that a dysregulation of the proteasomal pathways was not significant in STRING enrichment analysis. These findings could be interpreted as activated selective capacity for the vesicle-mediated elimination of mitochondrial fragments and other immune-response triggering bacteria by lysosomes. Indeed, among the GO terms Biological Process, there was a significant enrichment for “phagocytosis” (FDR = 0.0047), with upregulation of Mesdc2, Colec12, Lrp1, Rab22a, B2m, Itgb3, Cdc42ep2 versus downregulation of Mfge8, Axl, Thbs1, Rab31, Tgm2, Kif5b, Flnb, Anxa1, Anxa3, Myo1c, Cd302, Myh9, Gsn, and Vim. As expected during innate immune responses, these changes were accompanied by massive changes in the REACTOME pathway “extracellular matrix organization” (FDR = 3.27 × 10^−15^), in the KEGG pathway “focal adhesion” (9.94 × 10^−12^) and in the INTERPRO protein domains “growth factor receptor cysteine-rich domain superfamily” (1.54 × 10^−5^).

## 3. Discussion

This manuscript provides the first documentation of the global proteome of *LonP1*^+/−^ MEFs. While a strong effect on oxidative processes in the mitochondria was expected and did not require validation, novel observations included the lack of mitoribosomal accumulations despite Eral1 accumulation. The *LonP1*^+/−^ proteome profile of all mitochondrially localized factors showed Eral1 as second most significant upregulation effect (p = 1.97 × 10^−3^), after the Lonp1 heterozygous loss itself (p = 1.92 × 10^−4^) and the 2.3-fold accumulation of Abat (p = 9.25 × 10^−4^), as the crucial enzyme in the generation of succinate via breakdown of amino acids. The Eral1 increase was an unexpected finding, given that Eral1 was recently proposed as a ClpXP degradation substrate [47]. Eral1 is a mitochondrial matrix chaperone, which associates with the small ribosomal subunit and contributes to mitoribosome assembly [72]. Our novel proteome profile and two meta-analyses indicate that mitoribosome accumulation occurs only upon CLPX mutations and correlates with ClpX levels, while Eral1 accumulates upon Lonp1 mutation, ClpP loss or ClpX overexpression quite unspecifically.

In the bioinformatic analysis of the *LonP1*^+/−^ MEF global proteome, the previously published degradation substrates such as Aco2 and Tfam did not show changes in their protein abundances. Also, total mitochondrial biogenesis and mitophagy did not seem to be altered significantly, and proteasomal degradation was not activated. However, the lysosomal degradation of immunological targets was strongly upregulated together with phagocytosis and endosomal pathways, with the strongest upregulations concerning Ctsz and Ctsc which act in innate immune pathways [73,74]. Furthermore, it seemed credible that LonP1 deficiency affects the import of mitochondrial precursor proteins, since the mitochondrial import receptor Tomm70a, the import peptidase Pmpcb and the folding chaperone Hspd1 had significantly altered abundances. This is in good agreement with the previously established role of LonP1 in the unfolded protein response [75,76].

Several mitochondrial factors were significantly downregulated, which are involved in purine and pyrimidine synthesis, such as Mthfd1 and Dhodh, respectively [77,78]. This contrasted with the strong upregulation of Abat, which is known for its role in the conversion of dNDPs to dNTPs. These findings again hint towards an altered RNA and DNA homeostasis in LonP1-deficient cells. In *ClpP*^−/−^ mice, we previously observed an accumulation of mtDNA [46], whereas mtDNA was lower upon knockdown of LonP1 in B16F10 melanoma cells [12]. Both changes in the content of mitochondrial DNA are accompanied by alterations of the innate immune system. In the *ClpP*^−/−^ mice, upregulations occur for several factors that are important in Rig‑I signaling, which responds to viral RNA [46]. In *LonP1*-deficient cells, there were also upregulations of cytosolic factors responding to toxic RNA or DNA, such as Ifih1, Trim25, Mnda, and Ifi204. The observations in both *ClpP*^−/−^ and *LonP1*^+/−^ mouse mutants might reflect responses to the dysfunctional mitochondria and their altered state of nucleic acid content. It was recently shown that mitochondrial DNA or double-stranded RNA can escape to the cytosol and lead to cellular antiviral reactions [70,71]. Since both systems that lack a mitochondrial matrix protease show features of changes of the innate immune system, this may be a general mechanism with mitochondrial dysfunction as an underlying cause. In contrast to *ClpP*^−/−^ mice, the *LonP1*^+/−^ MEFs exhibited a significant downregulation of Trim44, which was shown to stabilize the mitochondrial antiviral signaling protein Mavs [57]. In regard to the lower amount of mtDNA upon LonP1 deficiency, this downregulation could be a response towards the upregulation of other cytosolic nucleic acid sensors, as an effort of the cell to minimize the inflammatory state. This innate immune system activation was also documented in another model of mitochondrial dysfunction, where mice with deletion of the mitochondrial transcription factor Tfam display a massive induction of inflammatory signatures [71].

In the analysis of the global proteome of *LonP1*^+/−^ MEF beyond mitochondria, mostly upregulations were detected for pathways involved in oxidative stress and glutathione. The strongest upregulation was seen for Catalase, a factor known to play a major role in oxidative stress responses [79]. The second highest upregulation was seen for Sod3, which is also established as a marker for oxidative stress [80]. Elevated levels also were observed for Mgst1, Oxr1 and many components of the glutathione pathway, which is involved in the neutralization of reactive oxidative species [81]. All these changes may reflect secondary cytoplasmic adaptations to altered oxidative processes in mitochondria.

Mitochondrial dysfunction is closely related to respiratory function. In chronically ClpP-deleted mice and cells, there are functional reductions in complex-I of the respiratory chain, but this might be an indirect effect upon chronic tissue pathology [46,47,82]. In the acute knockdown of ClpP or ClpX, respiratory function of complex-II was selectively reduced [82,83] and an accumulation of the subunits of complex-II was observed. In contrast, deficiency of LonP1 led to decreased activities of mitochondrial complex-I, III, and IV in heart tissue of LonP1 conditional knockout mice [84]. In LonP1^+/−^ mouse heart complex-I levels and activities were decreased [85]. Mutations of LonP1 in humans lead to a rare multi-system developmental disorder—CODAS syndrome. Patients show swollen mitochondria with abnormal inner membrane morphology and reduced respiratory capacity [12]. Our results revealed that *LonP1*^+/−^ MEF exhibited upregulations for three proteins in the HP membrane arm of complex-I (in contrast to *ClpP*-deleted tissues), namely Ndufa10, Ndufb7, and Ndufb4, whereas a slight downregulation was detected for one protein (Ndufa5) in the peripheral matrix arm (Q-module) [86]. Given that LonP1 is localized in the matrix, the accumulation of the membrane arm may be due to an indirect effect when a preassembled subcomplex consisting of the joint ND5, ND4, and ND2 module of the membrane arm cannot be docked onto an improperly folded Q module in the IP/FP matrix arm.

Lon protease function decreases with old age in mice. It was also seen that overexpression of the Lon protease orthologue in *P. anserina* resulted in a prolonged lifespan [30,87]. This hints towards its role in the regulation of stress and survival [88]. Mitochondrial mutants and altered life expectancy are intimately related and have been most studied in *C. elegans* [89]. The homozygous deficiency of LonP1 shows one of the most drastic effects on lifespan since mice die very early in utero and even the heterozygous depletion results in strong changes in various cellular processes. Here, we could show that there are similarities between the absence of ClpP and Lonp1, highlighting their crucial roles for protein homeostasis within mitochondria. But we also defined LonP1-deletion-specific consequences, so the relative targets of each protease may be inferred. Still, in order to understand all functions of ClpP and Lonp1, much future research will be needed.

## 4. Materials and Methods

### 4.1. Cell Culture

The LonP1^+/−^ MEF have been generated and described before [6]. Cells were maintained in Dulbecco’s minimal essential medium with 4.5 g/L glucose (Invitrogen, Karlsruhe, Germany) plus 15% fetal bovine growth serum (Gibco, One Shot, Schwerte, Germany), 1% Penicillin/Streptomycin (Gibco, Schwerte, Germany) and 1% Glutamine (Invitrogen, Karlsruhe, Germany) at 37 °C and 5% CO_2_ in a humidified incubator, and were passaged every 3–4 days. All cell lines were regularly tested for Mycoplasma contamination.

### 4.2. Proteomics

Protein abundance of cell pellets from *LonP1*^+/−^ MEF lines and their matching controls (n = 3, passage 4) were analyzed by label-free quantitative proteomics as recently described [90]. Mass spectrometry data were analyzed by Max Quant [91] and extended statistics were done with Perseus [92]. Quantified proteins were quality filtered for at least 3 label-free quantification values in one experimental group. Missing values were randomly replaced from normal distribution. Common contaminants and reverse identifications were excluded. For statistical comparison Student’s t-tests and permutation based False Discovery Rate with 250 randomizations were used.

### 4.3. Bioinformatic Analyses

For protein–protein-interaction (PPI) network analysis, the software tool STRING (Search tool for the retrieval of interacting genes) v.11.0 (https://string-db.org/) with standard settings was used to visualize networks among factors with nominally significant difference between HET and WT status, using three biological replicates [93]. Automated network statistics were performed; significant functional enrichments of GO (Gene Ontology terms regarding biological processes, molecular functions, cellular components), KEGG pathways, REACTOME pathways, PFAM protein domains, INTERPRO Protein Domains and Features, and SMART protein domains were exported into Excel files. Pathway findings were processed further into tables for dysregulated (*p*-values < 0.05) mitochondrial factors, oxidative stress, and glutathione factors, innate immunity factors as well as lysosomal factors.

## Figures and Tables

**Table 1:** List of mitochondrial factors with significant dysregulation, ordered by direction of change (dark green color for downregulations, red for upregulations) and by fold-changes. In response to the deficiency of the AAA+ domain containing peptidase LonP1 (purple), several AAA+ domain containing factors and proteases (highlighted in yellow), as well as two chaperones (orange), were altered. Several subunits of the respiratory complex-I stood out (green), with upregulations of three subunits in the membrane arm (P module) [60], while one subunit in the matrix arm of iron/sulfur and flavoproteins (NQ modules) showed a minor downregulation.

Protein Names	Gene Names	*p*-Value	q-Value	Fold Change
Lon protease homolog, mitochondrial	*Lonp1*	1.920 × 10^−4^	0.011	−1.921
Dihydroorotate dehydrogenase (quinone), mitochondrial	*Dhodh*	0.022	0.147	−1.438
Amine oxidase [flavin-containing] A	*Maoa*	0.008	0.102	−1.381
Solute carrier family 25 member 46	*Slc25a46*	0.044	0.355	−1.209
Mitochondrial-processing peptidase subunit beta	*Pmpcb*	0.012	0.290	−1.193
28S ribosomal protein S2, mitochondrial	*Mrps2*	0.042	0.379	−1.183
60 kDa heat shock protein, mitochondrial	*Hspd1*	0.004	0.251	−1.178
Voltage-dependent anion-selective channel protein 2	*Vdac2*	0.046	0.485	−1.123
NADH dehydrogenase [ubiquinone] 1 alpha subcomplex subunit 5	*Ndufa5*	0.031	0.576	−1.086
Mitochondrial import receptor subunit TOM70	*Tomm70a*	0.020	0.578	−1.081
C-1-tetrahydrofolate synthase,	*Mthfd1*	0.043	0.610	−1.078
Monofunctional C1-tetrahydrofolate synthase, mitochondrial	*Mthfd1l*	0.050	0.729	−1.051
				
4-aminobutyrate aminotransferase, mitochondrial	*Abat*	0.001	0.012	2.322
Calcium uptake protein 2, mitochondrial	*Micu2*	0.004	0.021	2.239
Monocarboxylate transporter 1	*Slc16a1*	0.002	0.021	1.718
Pyruvate carboxylase;Pyruvate carboxylase, mitochondrial	*Pcx;Pc*	0.007	0.087	1.428
Carnitine O-palmitoyltransferase 2, mitochondrial	*Cpt2*	0.003	0.075	1.393
Oxidation resistance protein 1	*Oxr1*	0.005	0.092	1.360
Mitochondrial chaperone BCS1	*Bcs1l*	0.013	0.157	1.320
Mitochondrial 2-oxoglutarate/malate carrier protein	*Slc25a11*	0.011	0.170	1.285
Nucleoside diphosphate-linked moiety X motif 8, mitochondrial	*Nudt8*	0.047	0.323	1.245
Propionyl-CoA carboxylase beta chain, mitochondrial	*Pccb*	0.004	0.164	1.242
Ferrochelatase;Ferrochelatase, mitochondrial	*Fech*	0.005	0.184	1.230
Probable arginine--tRNA ligase, mitochondrial	*Rars2*	0.023	0.295	1.217
AFG3-like protein 2	*Afg3l2*	0.013	0.272	1.206
NADH dehydrogenase [ubiquinone] 1 alpha subcomplex subunit 10, mitochondrial	*Ndufa10*	0.031	0.335	1.199
GTPase Era, mitochondrial	*Eral1*	0.002	0.216	1.185
Peptidyl-tRNA hydrolase 2, mitochondrial	*Ptrh2*	0.036	0.376	1.177
NADH dehydrogenase [ubiquinone] 1 beta subcomplex subunit 7	*Ndufb7*	0.018	0.380	1.149
NADH dehydrogenase [ubiquinone] 1 beta subcomplex subunit 4	*Ndufb4*	0.025	0.456	1.120
Calcium-binding mitochondrial carrier protein Aralar1	*Slc25a12*	0.016	0.443	1.119
Mitochondrial amidoxime reducing component 2	*Marc2*	0.019	0.452	1.118
Isocitrate dehydrogenase [NADP], mitochondrial	*Idh2*	0.049	0.519	1.109
Phosphoenolpyruvate carboxykinase [GTP], mitochondrial	*Pck2*	0.004	0.486	1.092

**Table 2:** List of dysregulated factors in the oxidative stress and antioxidant glutathione pathways, ordered by direction of change (red for upregulations, dark green for downregulations) and by fold-changes. The strongest upregulation was observed for the heme-iron-binding catalase (Cat), while another Cat peptide showed minor downregulation.

Protein Names	Gene Names	*p*-Value	q-Value	Fold Change
Catalase	*Cat*	0.012	0.021	7.218
Superoxide dismutase [Cu-Zn]; Extracellular superoxide dismutase [Cu-Zn]	*Sod3*	0.004	0.015	4.672
Aminopeptidase N	*Anpep*	0.001	0.000	3.443
Microsomal glutathione S-transferase 1	*Mgst1*	0.026	0.079	2.352
Glutathione S-transferase A4	*Gsta4*	0.012	0.048	2.016
Lactoylglutathione lyase	*Glo1*	0.043	0.155	1.653
Nicotinate phosphoribosyltransferase	*Naprt*	0.047	0.192	1.498
Glutathione S-transferase omega-1	*Gsto1*	0.001	0.047	1.415
Glutathione S-transferase Mu 5	*Gstm5*	0.036	0.197	1.391
Serine/threonine-protein kinase 24; Serine/threonine-protein kinase 24 35 kDa subunit; Serine/threonine-protein kinase 24 12 kDa subunit	*Stk24*	0.005	0.167	1.246
Isocitrate dehydrogenase [NADP]; Isocitrate dehydrogenase [NADP] cytoplasmic	*Idh1*	0.015	0.252	1.227
Maleylacetoacetate isomerase	*Gstz1*	0.012	0.295	1.190
Glutathione S-transferase Mu 2	*Gstm2*	0.004	0.294	1.160
Isocitrate dehydrogenase [NADP], mitochondrial	*Idh2*	0.049	0.519	1.109
Protein deglycase DJ-1	*Park7;Dj1*	0.021	0.509	1.098
				
Glutathione synthetase	*Gss*	0.015	0.229	−1.248
Spermine synthase	*Sms*	0.014	0.187	−1.286
Egl nine homolog 1	*Egln1*	0.029	0.297	−1.228
Catalase	*Cat*	0.022	0.318	−1.197

**Table 3:** List of dysregulated factors in the innate immune system, ordered by direction of change (red for upregulations, dark green for downregulations) and by fold-changes. Among the many existing receptors for damage-associated-patterns, components of the pathways for detection of toxic DNA (highlighted by orange color) and double-stranded RNA (yellow) with their downstream nuclear transcription factors in the Stat-family (sky blue) were prominent among the upregulations. Plasma membrane epitopes (light green color) within the cluster of differentiation CD* superfamily stood out among the downregulations.

Protein Names	Gene Names	*p*-Value	q-Value	Fold Change
Transmembrane glycoprotein NMB	*Gpnmb*	0.008	0.021	4.168
Interleukin-1 receptor antagonist protein	*Il1rn*	7.280 × 10^−7^	0.000	3.323
Osteopontin	*Spp1*	0.045	0.108	2.422
Lymphocyte antigen 6A-2/6E-1	*Ly6a*	0.008	0.033	2.235
Atypical chemokine receptor 3	*Ackr3*	0.002	0.021	1.919
Coxsackievirus and adenovirus receptor homolog	*Cxadr*	0.006	0.035	1.913
Interferon-activable protein 204	*Ifi204*	0.025	0.091	1.856
H-2 class I histocompatibility antigen, D-B alpha chain	*H2-D1;H-2D;H2-L*	0.013	0.057	1.820
H-2 class I histocompatibility antigen, K-K alpha chain;	*H2-K1;H2-K;H2-D1*	0.013	0.058	1.808
Tetraspanin;CD82 antigen	*Cd82*	4.349 × 10^−5^	0.014	1.799
Signal transducer and activator of transcription;Signal transducer and activator of transcription 2	*Stat2*	0.024	0.094	1.768
Interferon-induced 35 kDa protein homolog	*Ifi35*	0.037	0.125	1.767
Interferon-induced helicase C domain-containing protein 1	*Ifih1*	0.023	0.107	1.613
Tetraspanin;Tetraspanin-6	*Tspan6*	0.013	0.083	1.597
Gamma-interferon-inducible lysosomal thiol reductase	*Ifi30*	0.001	0.021	1.591
Signal transducer and activator of transcription;Signal transducer and activator of transcription 1	*Stat1*	0.027	0.136	1.529
Stimulator of interferon genes protein, STING	*Tmem173*	0.016	0.119	1.440
Interferon-induced, double-stranded RNA-activated protein kinase, PKR	*Eif2ak2*	0.021	0.155	1.395
Interferon-activable protein 205-B; Interferon-activable protein 205-A	*Ifi205b;Mnda; Ifi205a*	0.007	0.113	1.347
E3 ubiquitin/ISG15 ligase TRIM25	*Trim25*	0.011	0.138	1.345
Lymphocyte-specific protein 1	*Lsp1*	0.016	0.162	1.342
Interferon regulatory factor 2-binding protein 2	*Irf2bp2*	0.007	0.114	1.341
Interleukin-6 receptor subunit beta	*Il6st;il6st*	0.011	0.202	1.249
Signal transducer and activator of transcription; Signal transducer and activator of transcription 3	*Stat3*	0.026	0.374	1.167
				
Tripartite motif-containing protein 44	*Trim44*	0.002	0.014	−10.823
CD97 antigen	*Adgre5;Cd97*	0.002	0.050	−1.435
CD44 antigen	*Cd44*	0.012	0.105	−1.431
CD302 antigen	*Cd302*	0.041	0.255	−1.319
CD109 antigen	*Cd109*	0.001	0.081	−1.311
Poliovirus Receptor	*Pvr*	0.012	0.194	−1.262
Interferon regulatory factor 2-binding protein-like	*Irf2bpl*	0.016	0.283	−1.208

**Table 4:** List of dysregulated factors in the lysosomal compartment, ordered by direction of change (red for upregulations, dark green for downregulations) and by fold-changes.

Protein Names	Gene Names	*p*-Value	q-Value	Fold Change
Dipeptidyl peptidase 1; Dipeptidyl peptidase 1 exclusion domain chain; Dipeptidyl peptidase 1 heavy chain; Dipeptidyl peptidase 1 light chain	*Ctsc*	0.001	0.013	2.148
Transmembrane protein 59	*Tmem59*	0.016	0.080	1.783
Cathepsin Z	*Ctsz*	0.015	0.092	1.550
Alpha-mannosidase; Lysosomal alpha-mannosidase	*Man2b1*	0.010	0.082	1.530
Beta-hexosaminidase; Beta-hexosaminidase subunit alpha	*Hexa*	0.003	0.046	1.527
Sulfatase-modifying factor 1	*Sumf1*	0.033	0.157	1.508
Alpha-galactosidase A	*Gla*	0.012	0.094	1.484
Beta-glucuronidase	*Gusb*	0.003	0.053	1.470
Type 1 phosphatidylinositol 4,5-bisphosphate 4-phosphatase	*Tmem55b*	0.005	0.079	1.430
Cathepsin D	*Ctsd*	3.585 × 10^−4^	0.033	1.415
Prosaposin	*Psap*	0.021	0.149	1.413
Lysosomal thioesterase PPT2	*Ppt2*	0.046	0.228	1.399
Putative phospholipase B-like 2; Putative phospholipase B-like 2 28 kDa form; Putative phospholipase B-like 2 40 kDa form; Putative phospholipase B-like 2 15 kDa form	*Plbd2*	0.046	0.230	1.397
Carboxypeptidase; Lysosomal protective protein; Lysosomal protective protein 32 kDa chain; Lysosomal protective protein 20 kDa chain	*Ctsa*	0.048	0.233	1.393
Granulins; Acrogranin; Granulin-1; Granulin-2; Granulin-3; Granulin-4; Granulin-5; Granulin-6; Granulin-7	*Grn*	0.017	0.144	1.388
N-Acetyl-Alpha-Glucosaminidase	*Naglu*	0.007	0.093	1.387
Lysosomal alpha-glucosidase	*Gaa*	0.007	0.099	1.385
Arylsulfatase B	*Arsb*	0.024	0.170	1.376
WD repeat-containing protein 59	*Wdr59*	0.011	0.120	1.375
Beta-hexosaminidase; Beta-hexosaminidase subunit beta	*Hexb*	0.015	0.148	1.366
Glucosylceramidase	*Gba*	0.006	0.097	1.360
Gamma-glutamyl hydrolase	*Ggh*	0.011	0.133	1.355
Beta-galactosidase	*Glb1*	0.004	0.093	1.343
Cation-independent mannose-6-phosphate receptor	*Igf2r*	0.002	0.087	1.326
Ganglioside GM2 activator	*Gm2a*	4.257× 10^−4^	0.053	1.318
Lysosomal Pro-X carboxypeptidase	*Prcp*	0.031	0.286	1.249
Transmembrane protein 106B	*Tmem106b*	0.029	0.281	1.248
Dipeptidyl peptidase 2	*Dpp7*	0.022	0.268	1.238
Beta-mannosidase	*Manba*	0.022	0.274	1.232
N(4)-(beta-N-acetylglucosaminyl)-L-asparaginase; Glycosylasparaginase alpha chain; Glycosylasparaginase beta chain	*Aga*	0.012	0.323	1.171
V-type proton ATPase subunit H	*Atp6v1h*	0.038	0.394	1.167
Ragulator complex protein LAMTOR1	*Lamtor1*	0.040	0.443	1.140
AP-1 complex subunit beta-1; AP complex subunit beta	*Ap1b1*	0.018	0.411	1.134
				
Pro-cathepsin H; Cathepsin H mini chain; Cathepsin H; Cathepsin H heavy chain; Cathepsin H light chain	*Ctsh*	8.684× 10^−6^	0.000	−1.734
Sequestosome-1	*Sqstm1*	0.009	0.212	−1.232

## Supplementary Materials

Supplementary materials can be found at https://www.mdpi.com/1422-0067/20/18/4523/s1.

## Abbreviations

AAA+	ATPases Associated with diverse cellular Activities
AFG3L2	AFG3-Like matrix AAA peptidase subunit 2
ATPase	Adenosine Tri-Phosphatase
CLPP	Caseinolytic Mitochondrial Matrix Peptidase Proteolytic Subunit
CLPX	Caseinolytic Mitochondrial Matrix Peptidase Chaperone Subunit
CO2	Carbon dioxide
CoA	Coenzyme-A
CODAS	Syndrome with cerebral, ocular, dental, auricular, and skeletal anomalies
DNA	Deoxyribonucleic acid
dNDPs	Deoxy-Nucleoside Di-Phosphate
dNTPs	Deoxy-Nucleoside Tri-Phosphate
dsRNA	Double-stranded RNA
FDR	False discovery rate
g	gram
GABA	Gamma-Amino Butyric Acid
GO	Gene Ontology
HET	Heterozygous mutant
HP arm	Membrane hydrophobic part of respiratory complex I
INTERPRO	Database of protein families, domains and functional sites
IP/FP arm	Iron-sulfur-cluster containing part and FMN containing part of respiratory complex I
KEGG	Kyoto Encyclopedia of Genes and Genomes
KO	knockout
l	liter
LC3	Light Chain 3 protein, encoded by the *MAP1LC3A* gene
LIM-type	Zinc finger domain named after proteins LIN-11, Isl1 and MEC-3 where they were first found
LONP1	Mitochondrial ATP-Dependent Protease Lon, or PRSS15 for Serine Protease 15
MCU	Mitochondrial Calcium Uniporter
MEF	Murine embryonal fibroblasts
mAAA	Matrix AAA Peptidase
mtDNA	Mitochondrial DNA
NOD	Nucleotide-binding oligomerization domain-containing protein
PARK2	Autosomal recessively inherited Parkinson’s disease type 2, caused by mutations in Parkin
PARK6	Autosomal recessively inherited Parkinson’s disease type 6, caused by mutations in PINK1
PARK7	Autosomal recessively inherited Parkinson’s disease type 7, caused by mutations in DJ-1
PARL	Presenilin-Associated Rhomboid-Like protein, mitochondrial protease
PFAM	Database of protein families including their annotations and multiple sequence alignments
PINK1	PTEN-induced Kinase 1, mitochondrial
PRLTS3	Perrault syndrome type 3 due to CLPP mutations
REACTOME	Database of reactions, pathways and biological processes
RIG-I	Retinoic Acid-Inducible Gene 1 Protein, encoded by the *Ddx58* gene
RNA	Ribonucleic acid
SCA28	Spinocerebellar Ataxia type 28, caused by mutations in
SMART	Simple Modular Architecture Research Tool
SMDT1	Single-pass Membrane protein with Aspartate-rich Tail 1, mitochondrial
STING	Stimulator of INterferon Genes protein, encoded by the *Tmem173* gene
STRING	Search tool for the retrieval of interacting genes
TCA cycle	Tricarboxylic acid cycle, Citric acid cycle, Krebs cycle
UPRmt	Mitochondrial Unfolded Protein Response
WT	Wildtype
