# Supplementary material for: Global Proteome of LonP1+/− Mouse Embryonal Fibroblasts Reveals Impact on Respiratory Chain, but No Interdependence between Eral1 and Mitoribosomes
Source: Int J Mol Sci. 2019 Sep 12;20(18):4523. doi: 10.3390/ijms20184523 (PMC6770551; doi:10.3390/ijms20184523)
Supplement: Supplementary file 1 [file ijms-20-04523-s001.zip › ijms-557919 supplementary done/figures and tables' caption.pdf]

Supplementary Table S1. ***LonP1*$^{+/-}$ mouse embryonal fibroblast global proteome profile via label-free mass spectrometry.** The data sheet shows imputed Perseus output data (where significant upregulations are shown in red color, significant downregulations in green, the mutant protein in blue) and a list of detected peptides with relevant measures. Significance is scored as a nominal variant in column F, significant upregulations are highlighted in column G, significant downregulations in column H, these proteins were analyzed further in STRING bioinformatics. Fold-changes are documented in column D, p-values in column E, q-values in column I.

Supplementary Table S2. **STRING analysis of enriched protein-protein interactions within the previously published global proteome profile of C2C12 myoblasts with ClpX overexpression** [36]. Different datasheets present automated bioinformatics on defined (i) KEGG pathways, (ii) GO-terms Cellular Component, (iii) GO terms Biological Process, (iv) GO terms Molecular function, assigning statistical significance via false discovery rates (FDR values) in column D and identifying the dysregulated factors within each pathway in column F.

Supplementary Figure S1. **Global proteome profile of *ClpX*-overexpressing myoblast cells.** STRING diagram of interactions among proteins with >1.5-fold accumulation in myoblasts with ClpX overexpression, as bioinformatics re-assessment of a dataset published previously [36]. Mitochondrial proteins are identified by bullets in red color. Prominent clusters reflect the accumulation of ClpX with mitochondrial Hspa9 (1.5-fold), Hspd1 (1.8-fold) and several extra-mitochondrial chaperones (highlighted by circle with pink background), as well as the accumulation of mitoribosomal proteins (between 2-fold and 1.1-fold, circle with green background), of proteins in the nucleolus as a pre-ribosome factory (circle with orange background), of proteins in the nuclear pore and in microtubule transport (circle with grey background) and of proteins within the mitochondrial import pore (clustered above and within the yellow circle).
