# Supplementary figures and images for: Global Proteome of LonP1+/− Mouse Embryonal Fibroblasts Reveals Impact on Respiratory Chain, but No Interdependence between Eral1 and Mitoribosomes
Source: Int J Mol Sci. 2019 Sep 12;20(18):4523. doi: 10.3390/ijms20184523 (PMC6770551; doi:10.3390/ijms20184523)

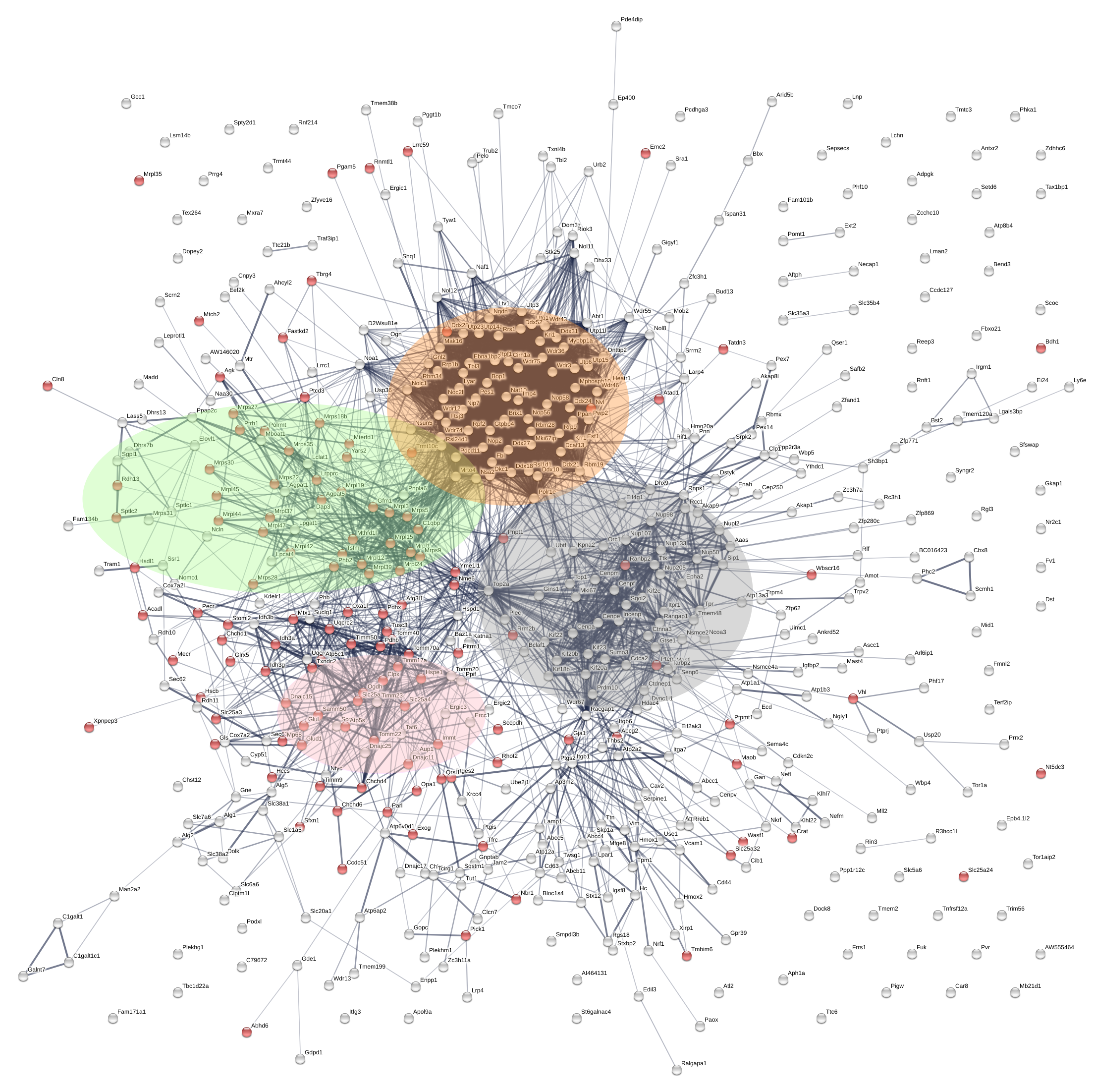

Supplement: Supplementary file 1 [file ijms-20-04523-s001.zip › ijms-557919 supplementary done/KeyAuburger_SupplFigA1_CLPXoverexpr_1.5foldUp_RibosomeBiogenesis_string.tif]
